# Temporal Variability and Social Heterogeneity in Disease Transmission: The Case of SARS in Hong Kong

**DOI:** 10.1371/journal.pcbi.1000471

**Published:** 2009-08-21

**Authors:** Anne Cori, Pierre-Yves Boëlle, Guy Thomas, Gabriel M. Leung, Alain-Jacques Valleron

**Affiliations:** 1 INSERM, Paris, France; 2 Université Pierre et Marie Curie-Paris6, Paris, France; 3 School of Public Health, Li Ka Shing Faculty of Medicine, The University of Hong Kong, Hong Kong, Special Administrative Region, People's Republic of China; Imperial College London, United Kingdom

## Abstract

The extent to which self-adopted or intervention-related changes in behaviors affect the course of epidemics remains a key issue for outbreak control. This study attempted to quantify the effect of such changes on the risk of infection in different settings, i.e., the community and hospitals. The 2002–2003 severe acute respiratory syndrome (SARS) outbreak in Hong Kong, where 27% of cases were healthcare workers, was used as an example. A stochastic compartmental SEIR (susceptible-exposed-infectious-removed) model was used: the population was split into healthcare workers, hospitalized people and general population. Super spreading events (SSEs) were taken into account in the model. The temporal evolutions of the daily effective contact rates in the community and hospitals were modeled with smooth functions. Data augmentation techniques and Markov chain Monte Carlo (MCMC) methods were applied to estimate SARS epidemiological parameters. In particular, estimates of daily reproduction numbers were provided for each subpopulation. The average duration of the SARS infectious period was estimated to be 9.3 days (±0.3 days). The model was able to disentangle the impact of the two SSEs from background transmission rates. The effective contact rates, which were estimated on a daily basis, decreased with time, reaching zero inside hospitals. This observation suggests that public health measures and possible changes in individual behaviors effectively reduced transmission, especially in hospitals. The temporal patterns of reproduction numbers were similar for healthcare workers and the general population, indicating that on average, an infectious healthcare worker did not infect more people than any other infectious person. We provide a general method to estimate time dependence of parameters in structured epidemic models, which enables investigation of the impact of control measures and behavioral changes in different settings.

## Author Summary

Recent epidemics have shown that healthcare workers may be overrepresented among cases and how critical it is to protect them. For example, during the 2002–2003 severe acute respiratory syndrome (SARS) epidemics in Hong Kong, 27%of cases were healthcare workers when they were <1% of the population. Better means of protection require understanding how healthcare workers were infected and assessing their role in disease transmission. Here, we describe a method for estimating the temporal profile of the risk of infection and probability of transmission in the community and hospitals. The 2002–2003 SARS outbreak in Hong Kong is used as an example. For the SARS epidemic, we show that the risk of infection in the community and hospitals decreased with time down to zero in hospitals but remained larger in the community. This observation suggests that public health measures and behavioural changes most effectively reduced transmission in hospitals. Besides, we find that the large number of cases observed among healthcare workers is more likely a result of large and sustained exposure to hospitalized cases than to transmission among healthcare workers. These results are of interest to design control measures in the event of an influenza pandemic.

## Introduction

Emerging infectious diseases have been defined as, “infections that have newly appeared in a population or have existed previously but are rapidly increasing in incidence or geographic range. [1]” Several features may make them particularly threatening. First, recognizing the disease can be difficult when the first cases appear, especially when the symptoms are non-specific. Second, no vaccine or specific treatment may be known initially. Moreover, heterogeneities in disease transmission may create high-risk groups, such as healthcare workers [2]–[5] and high-risk geographical areas, thereby dramatically enhancing the impact of the outbreak [6].

The 2003 severe acute respiratory syndrome (SARS) outbreak in Hong Kong is remarkably illustrative of the above issues: symptoms were similar to pneumonia [7]; the incubation period was long enough for local and international transmission to occur [8]; no vaccine or treatment was available; as much as 21% of cases worldwide were healthcare workers [9]. The outbreak also demonstrated the possible existence of super-spreading events (SSEs) [10], during which a few infectious individuals contaminated a high number of secondary cases. Hong Kong had two SSEs: the first occurred in Hospital X around March 3 and led to about 125 cases [11]; the second occurred in Housing Estate Y on March 19, and led to over 300 cases [12],[13]. Despite its particularly threatening features, the outbreak was brought under control.

In this context, once the epidemic is detected, spontaneous changes in behavior will occur, and non-pharmacological measures are usually initiated to control the outbreak. The resulting effects of these two phenomena on disease transmission is not easily quantified.

The effective contact rate, which reflects the combined influences of social proximity (the number of contacts per time unit) and the probability of infection through each contact, is an essential determinant of disease spread. Our aim was to estimate the temporal variation of this parameter in the community and hospitals, over the course of the outbreak.

Previously published mathematical models of parameter estimation addressed the issues of temporal variability [12],[14] or social heterogeneity [2],[15]. Here we present an approach that deals with both issues, together with the occurrence of SSEs. Then the method is applied to the 2003 SARS epidemic in Hong Kong (SARSID database [13]).

## Materials and Methods

### Data

Among the 1755 patients admitted to Hong Kong hospitals in 2003 for suspected SARS, 1467 serologically confirmed SARS cases were retained for analysis. For each case, occupation, date of symptom onset, date of hospital admission, duration of hospital stay and discharge status (dead or alive) were recorded. Durations of hospital stay were missing for 12 cases and imputed to 100 days.

### Transmission Model

**Figure 1:**
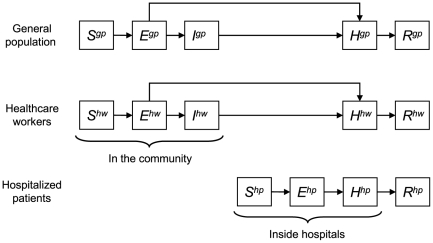
Compartmental Model for SARS Transmission in Hong Kong. Superscript letters denote social categories: 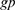, general population; 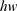, healthcare workers; 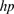, hospitalized patients. Disease states are: 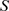, susceptible; 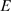, exposed (infected but not yet infectious); 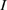, infectious not hospitalized; 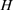, infectious hospitalized, and 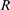, removed (recovered or dead).

The epidemic process was cast into a discrete time stochastic susceptible-exposed-infectious-removed (SEIR) compartmental model, designed to reflect a two-way classification of individuals according to disease status and ‘social’ category (Figure 1).

The latter was defined in three categories: hospitalized patients (hp), healthcare workers (hw), and the general population (gp).

**Figure 2:**
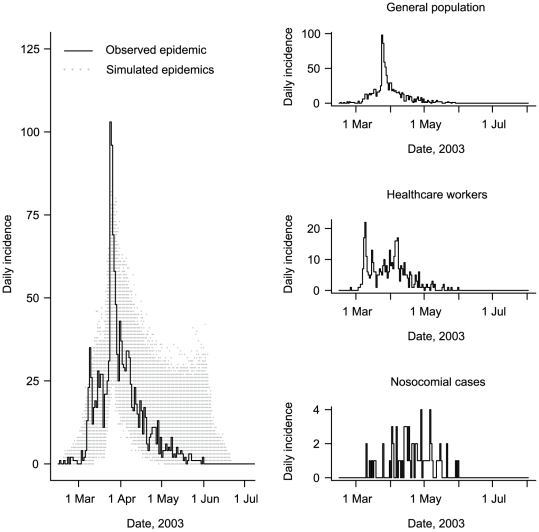
Daily Incidence of SARS Symptom Onset (Observed and 5×10^4^ Simulated Epidemics), Hong Kong, 2003. Cases were defined as: nosocomial when patients had been hospitalized for ≥5 days before symptom onset 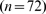; healthcare workers when they were indeed healthcare workers and not nosocomial 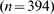; and general population, otherwise 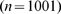. The grey cloud surrounding the observed epidemic curve corresponds to simulated epidemic curves.

According to these three social categories, SARS cases were qualified: nosocomial when the patient had been hospitalized for ≥5 days before symptom onset 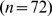; healthcare workers when the subjects were indeed healthcare workers and not nosocomial 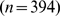; or general population, all others 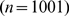. Their corresponding epidemic curves are shown in Figure 2.

Disease status was described in five compartments: susceptible (S), exposed (E), infectious non-hospitalized (I), infectious hospitalized (H), and removed (R). Individuals are initially susceptible to the disease and infected through contact with infectious subjects. Once infected, individuals are first exposed (infected, non-infectious) and then become infectious. The infectious stage is defined as the period of time during which infectious individuals can transmit the disease through contact with susceptibles. Finally, the infectious individuals are removed, either through recovery or death. Quarantine or isolation was not documented in the database, and was not specifically described: possibly isolated infectious individuals remain in stage 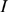 or 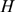, and quarantined contacts remain in stage 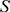.

Thus, depending on social category, susceptible individuals may be in compartments 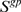 (general population), 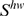 (healthcare workers), or 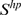 (hospitalized patients); similarly, exposed and recovered individuals may be in compartments 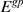, 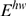 or 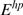, and 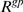, 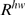 or 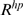, respectively; while infectious subjects are in compartments 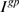 or 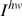 before hospitalization, and in compartments 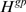, 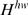 or 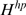 once hospitalized.

The size of the Hong Kong population (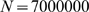) was obtained from local census data (http://www.info.gov.hk/info/hkbrief/eng/living2.htm). The number of hospitalized patients (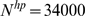) equaled the number of hospital beds in Hong Kong (http://www.info.gov.hk/info/hkbrief/eng/living2.htm). The number of healthcare workers (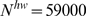) was derived from the healthcare worker-to-bed ratio in the Hospital X [13]. 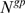, 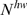 and 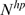 were assumed to be constant throughout the epidemic. Under this steady-state assumption, transitions between compartments 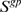, 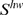, and 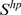 did not have to be included explicitly in the model.

The model assumes that there is no direct contact between hospitalized individuals and non hospitalized individuals from the general population. In particular, susceptible individuals in the general population (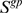 compartment) cannot be infected by infectious hospitalized SARS cases (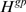, 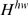, and 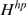 compartments), and susceptible hospitalized patients (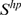 compartment) cannot be infected by infectious not-yet-hospitalized cases from the general population (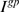 compartment).

### Statistical Model

In the following, **1**_{.}_ denotes the indicator function, defined by 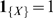 if 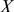 is true, and 0 otherwise.

For each Hong Kong inhabitant 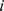 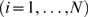, let 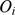 be the time of symptom onset, 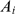 the day of hospital admission, 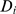 the day of hospital discharge, 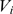 the day of death (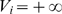 if the case did not die from SARS), and 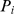 the social category (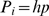 if 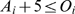, 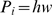 if 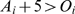 and inhabitant 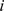 is a healthcare worker, and 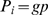 otherwise). For all inhabitants who were not infected by SARS, we let 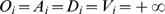.

For each individual 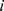 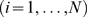, let 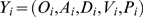. The observed data 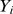 were augmented with 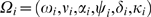, where 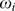, 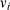, 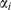 and 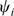 correspond to the dates of transition into the 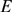, 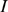, 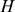 and 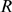 states respectively ; 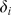 is the date of death, and 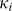 is the social category for case 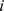 (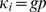, 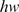 or 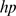).

Letting 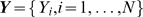 and 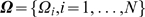, the joint density 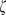 of 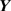, 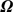, and of the vector 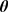 of unknown parameters is written as the following product:
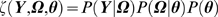
where 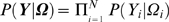, and 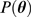 is a prior distribution for 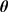.

As defined by Auranen et al. [17], 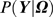, 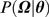 and 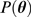 refer to the observation level, the transmission level and the prior level respectively.

The observation level ensures that the observed data are consistent with the augmented data.

During the SARS outbreak, few cases were reportedly infected by asymptomatic persons, but cases rapidly became infectious after symptom onset [12],[18],[19]. Therefore, for each case 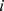, the onset of symptom was considered acceptable if 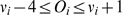.

The day 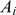 of hospital admission was consistent with the augmented data if 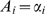 when the case was infectious prior to hospitalization (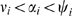) 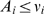 when the case was infectious only after hospitalization (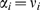) 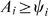 when the case was not infectious anymore at the time of hospitalization (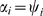).

It was also assumed that the infectious period did not outlast hospital discharge, that is 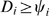


The date of death was 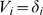.

Finally, the professional category 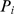 was acceptable if 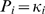.

Hence:
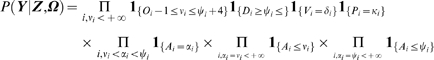



The transmission level describes SARS transmission, assuming 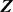 and 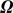 are known, conditional on the day 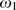 of infection of the first case.

A deterministic latent period of 5 days was assumed for all cases (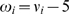 for 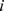 such that 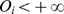) [13].

The duration of the infectious period (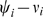) for SARS cases was gamma-distributed, with mean 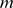 and variance 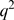. We let 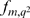 and 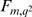 denote its density and cumulative distribution function respectively. For SARS patients dead on discharge, the infectious period was considered censored by death. Since the infectious period was defined as the period during which infectious cases can transmit the disease through contact with susceptibles, its distribution was assumed to remain the same over the course of the epidemic.

The specific stochastic infection rates on day 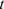 for susceptible individuals in compartments 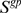, 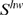, and 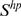 are: 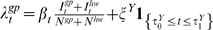, 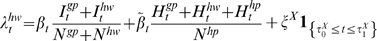 and 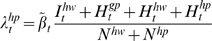, where 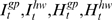 and 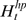 denote the numbers of individuals in compartments 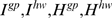 and 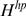, respectively; 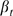 and 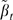 are the daily effective contact rates in the community and hospitals, respectively; 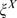 and 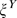 are temporary level shift interventions [4] reflecting the increment of infectiousness during the Hospital X and Housing Estate Y SSEs, i.e. from days 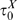 and 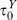 to days 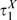 and 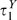.

This leads to:
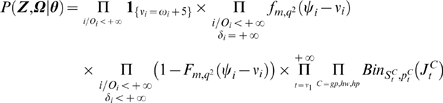
where 
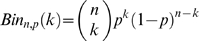
; 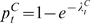 (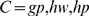), and 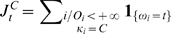 (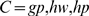) is the incidence in 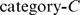 on day 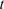.

The vector 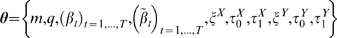 comprised about 228 unknown parameters, the epidemic lasting about 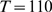 days.

For all model parameters except the effective contact rates, independent prior distributions were chosen. For the time of start of SSEs, the prior distributions were informative (see Table 1). The effective contact rates 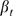 and 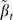 were modeled as second-order Gaussian random walks, on the log scale, with flat exponential priors on the first two states of the random walk. In this approach, the respective variances 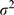 and 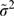 of innovations correspond to the smoothing parameters of cubic smoothing splines [21]; smaller values of 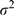 and 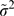 are associated with smoother trajectories. For the two precision parameters 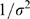 and 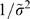, exponential hyperpriors with mean 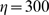 were selected. A sensitivity analysis of the hyperparameter value was performed (see Text S1).

**Table 1:** Prior Distributions for Model Parameters.

Parameters	Description	Prior Distributions
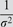 , 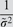	Inverse variance of innovations of random walks	Exponential distribution (mean 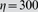 )
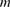 , 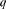	Mean and standard deviation of the infectious period	Gamma distribution (mean 200, variance 40000)
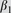 , 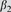	Effective contact rates in the community during the first two days	Exponential distribution (mean 1000)
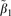 , 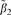	Effective contact rates in hospitals during the first two days	Exponential distribution (mean 1000)
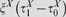 , 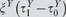	Area under SSE curves	Exponential distribution (mean 1000)
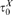	Day Hospital X SSE starts	Uniform distribution on 02/18–03/10
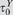	Day Housing Estate Y SSE starts	Uniform distribution on 03/14–03/24
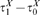 , 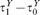	SSE durations	Poisson distribution (mean 3)

### Parameter Estimation

A Markov chain Monte Carlo (MCMC) method was used to sample the joint posterior distribution 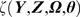 [22],[23]. More details on the sampler are provided in Text S2.

From the joint posterior distribution of the parameters, a number of meaningful epidemiological quantities, such as daily case-reproduction numbers [24] in each category (see Text S3), could be derived. In particular, the number of cases generated by each SSE could be estimated.

## Results

Estimates of the days of SSE starts and ends, increments (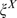, 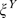), and the number of SSE cases in Hospital X and Housing Estate Y are shown in Table 2. Despite the somewhat shorter SSE duration for Housing Estate Y than for Hospital X, 2.5 times more cases occurred in Housing Estate Y than Hospital X.

**Table 2:** Estimated Parameters for Super Spreading Events in Hospital X and Housing Estate Y.

Site	Parameter	Unit	Mean	95% Credible Intervals
Hospital X	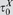		March 1, 2003	February 28–March 2, 2003
	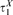		March 11, 2003	March 10–March 13, 2003
	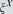	Days^−1^	1.4×10^−4^	(1.1×10^−4^–1.8×10^−4^)
	Size	Cases	94	(72–118)
Housing Estate Y	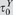		March 18, 2003	March 17–March 18, 2003
	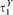		March 23, 2003	March 23–March 24, 2003
	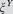	Days^−1^	5.2×10^−6^	(4.1×10^−6^–6.4×10^−6^)
	Size	Cases	235	(185–288)

**Figure 3:**
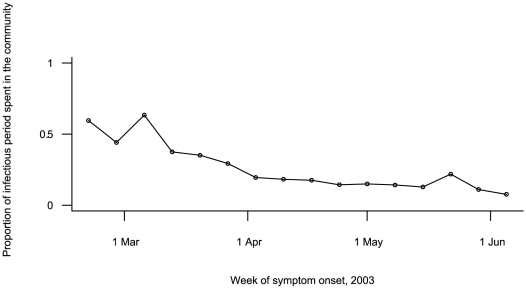
Proportion of Infectious Period Spent in the Community before Hospitalization as a Function of Week of Symptom Onset.

The estimated mean of the infectious period was 9.3 days (95% credible interval: (8.6–9.9)), with an estimated standard deviation of 2.3 days (95% credible interval: (1.8–2.9)). The proportion of the infectious period spent in the community decreased continuously with time (>60% at the beginning, <20% as early as early April). Toward the end of the epidemic, >95% of the infectious period was spent inside hospitals (see Figure 3).

**Figure 4:**
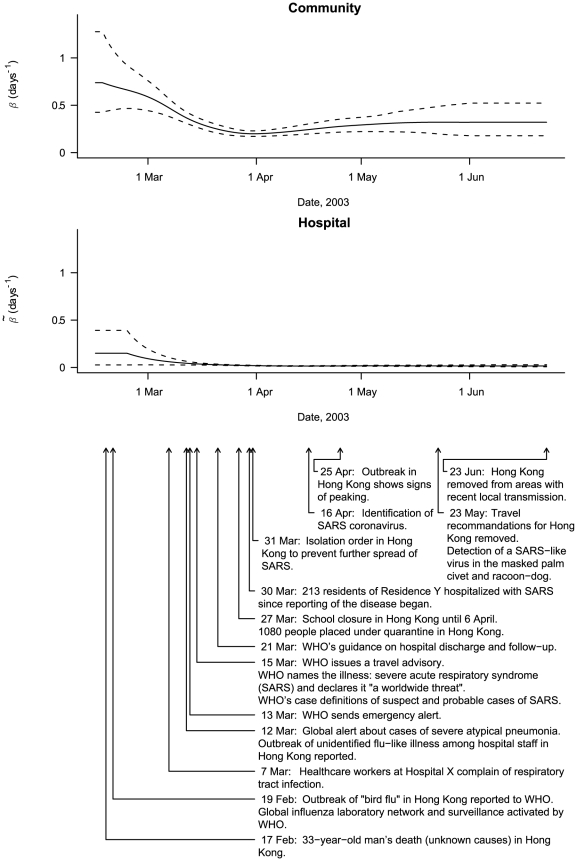
Mean Effective SARS Contact Rates (solid line) and 95% Credible Intervals (dashed line) in the Community and Hospitals as a Function of Time and Dates of Important Events.

The daily effective contact rates in the community (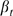) and hospitals (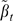) exhibited progressive a decrease in time, as shown in Figure 4. However, while the contact rate was almost 0 by late March inside hospitals, it remained >0.17 in the community.

The case-reproduction number first increased to 5.1 in the general population in late February and to 3.0 for healthcare workers in early March (see Text S3). It then decreased until the end of the epidemic. The case-reproduction numbers was <1 on March 13 for healthcare workers and on March 20 for the general population. Among nosocomial cases, the case-reproduction number was always <1, with a maximum value of 0.2 on March 14.

The model's ability to reproduce the main features of the epidemic was checked by simulating 5000 epidemics with parameters sampled from the estimated joint posterior distribution, as described in Text S4. The size and duration of simulated epidemics, as well as cases breakdown in categories (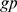, 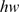, 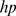) mirrored the Hong Kong epidemic (see Figure 2).

We also simulated 100 epidemics with a single set of parameters, sampled from the posterior distribution. Then, the estimation procedure was applied to each simulated epidemic in order to reestimate the parameters. The original parameters were in the estimated corresponding 95% credible interval in 87% of cases.

## Discussion

To rapidly and economically design and assess control measures for epidemics in modern societies, added insight into the dynamics of disease transmission is needed. These dynamics are conveniently summarized by critical, albeit non-observable, characteristics, such as the duration of the infectious period and effective contact rates. Estimation of these parameters from the observed data requires the development of mathematical models. Herein, we presented a model for epidemics that provides for social heterogeneity and time variability of transmission parameters. As a working example, the model was applied to the 2003 SARS epidemic in Hong Kong.

The effect of interventions and/or changes in behavior during the 2003 SARS outbreak may be modelled as time varying contact rates [12],[15],[25] or involve shortening of the infectious period [19]. Here, we adopted the first view. To assess if the data supported this choice, a model was fit where, in addition to time varying contact rates, we allowed the mean infectious period to change over three consecutive periods. The three posterior means were 9.5 days (before March 20), 9.2 (March 21 to April 9) and 10 days (after April 10), indicating that the time varying contact rates alone model the data adequately.

While the duration of the infectious period is an obvious determinant of disease transmission, no estimate has been available for SARS. The distribution of the viral load was found to peak 8–10 days after symptom onset [13],[18],[26],[27]. Here, assuming that the infectious period started between 1 day before and 4 days after symptom onset, it was estimated to extend over an average period of 9.3 days. We also found that the proportion of time infectious people spent outside hospitals decreased during the outbreak and was <5% at the very end, in agreement with Anderson et al. [18] and Leung et al. [13] who showed that the time from symptom onset to admission was shorter at the end of the epidemic.

One of the most striking features of the Hong Kong SARS epidemic was the occurrence of two SSEs. By definition, SSEs correspond to exceptional circumstances that are usually limited to well-circumscribed areas, such as Hospital X and Housing Estate Y, and last for only a few days [10]. In this respect, the very high contact rates generated by the SSEs were modeled as ‘innovation outliers’ [28], to avoid spurious overestimation of contact rates among the Hong Kong population.

Whether SSEs are a result of a few particularly highly infectious cases (excreting much virus and/or highly connected socially), or of particular environmental circumstances, or maybe both, remains unclear [16],[29],[30]. In our model, the force of infection associated with each SSE was independent on the number of currently infectious cases. The duration of SSEs was estimated independently for each SSE, and was independent on the duration of the infectious period. Therefore, our model was consistent with all possible causes of SSEs: one or several super-spreaders, or particular environmental circumstances, etc.

The level shift interventions [20] that were superimposed on the process describing the time evolution of the infection rates differed significantly from zero. Taking into account only serologically confirmed cases, we estimated that the Hospital X SSE began on March 1^st^, lasted 11 days and was responsible for 94 cases; and that the Housing Estate Y SSE began on March 18, lasted 6 days and caused 235 cases. Previous studies investigating SSEs in Hong Kong used all cases. By contact tracing, Lee et al. [11] found that the Hospital X SSE started on March 4 and involved 125 cases; the Housing Estate Y SSE had been estimated to start on March 19 [13] and to involve 312–330 [13] or 331 [12] cases.

Effective contact rates were estimated on a daily basis, in the community and hospitals. Both rates tended to decline, probably reflecting the effect of control measures (listed in Figure 4 [31],[32]) or self-adopted behavioral changes. The measures seem to have been particularly effective in hospitals, where the effective contact rate was 0 by late March, whereas the risk in the community did not decrease as sharply. In both settings, the effective contact rate was almost constant after late March, when no more control measures were introduced.

Others who studied the dependence of disease transmission on time reported reproduction numbers rather than effective contact rates [12],[14]. While the daily effective contact rates are sensitive to short-term day-to-day variations in transmission, the reproduction numbers reflect the integrated influences of the temporal evolution of effective contact rates, the infectious period duration and other factors, such as time spent in the community before hospitalization. Here, estimates of daily reproduction numbers were obtained for each social category. Notably, unlike Cauchemez et al. [14], it was not necessary to assume prior knowledge or constancy of the generation interval. The reproduction numbers showed a trend similar to the effective contact rates, with a clearly decreasing trend over time, suggesting that the epidemic was under control as early as mid-March (see Figure in Text S3). Moreover, the temporal patterns for the general population and healthcare workers were similar, with the reproduction numbers being higher for the general population, thereby indicating that on average, an infectious healthcare worker did not infect more people than any other infectious person. The reproduction numbers for nosocomial cases were much lower, either because they had fewer contacts or because the people they were in contact with were protected (typically healthcare workers wearing masks).

Our estimation procedure, applied to a set of 100 simulated epidemics, showed that in 87% of cases, the parameters used for simulation were inside the corresponding posterior 95% credible intervals. While most parameters were well estimated, the procedure tended to overestimate the duration of each SSE, while simultaneously underestimating its strength. The number of people affected by each SSE (i.e. population×duration×strength) was therefore correct, but its extent in time less robust. Ignoring the 17 days corresponding to both SSEs, 98% of the remaining parameters used for simulation were inside the posterior corresponding 95% credible intervals, indicating very little bias in our estimation procedure.

Herein, we described an approach to estimate the role of time variability and social heterogeneity in epidemic dynamics. Our model's simplifying assumptions such as the fixed duration of the latency period or the constant probability of transmission throughout the infectious period of cases, can be relaxed at the price of increasing complexity. Similarly, a more detailed model taking into account household transmission, and transmission inside and between hospitals, rather than assuming homogeneous mixing in the community and in hospitals, could be implemented, at the cost of a dramatic increase in the number of model parameters. More generally, the model can be easily accommodated to fit the specificities of any transmissible disease.

## Supporting Information

Text S1 Choice of Hyperparameter *η*
(0.09 MB PDF)

Text S2 Sampler Used for Parameter Estimation
(0.07 MB PDF)

Text S3 Estimation of the Case-Reproduction Number *R^A→B^_t_*
(0.39 MB PDF)

Text S4 Epidemic Simulation
(0.04 MB PDF)
