# Supplementary material for: Temporal Variability and Social Heterogeneity in Disease Transmission: The Case of SARS in Hong Kong
Source: PLoS Comput Biol. 2009 Aug 21;5(8):e1000471. doi: 10.1371/journal.pcbi.1000471 (PMC2717369; doi:10.1371/journal.pcbi.1000471)
Supplement: Text S1 — Choice of Hyperparameter η (0.09 MB PDF) [file pcbi.1000471.s001.pdf]

Text S1. Choice of Hyperparameter η

The following $\eta-$values were tested: 1, 5, 10, 50, 100, 200, 300, 400, 500, 10^3, 5×10^3, 10^4, and 2×10^4. For each value, model parameters were estimated, and 5×10^4 epidemics were simulated with parameters drawn from the joint posterior distribution. The daily incidence of symptom onset in each social category was recorded, and the sum of squared errors (between the incidence in observed and simulated epidemics) over days and social categories was taken as an indicator of goodness of fit of the model (see Figure S1.1). The $\eta-$ value minimizing this indicator was selected.

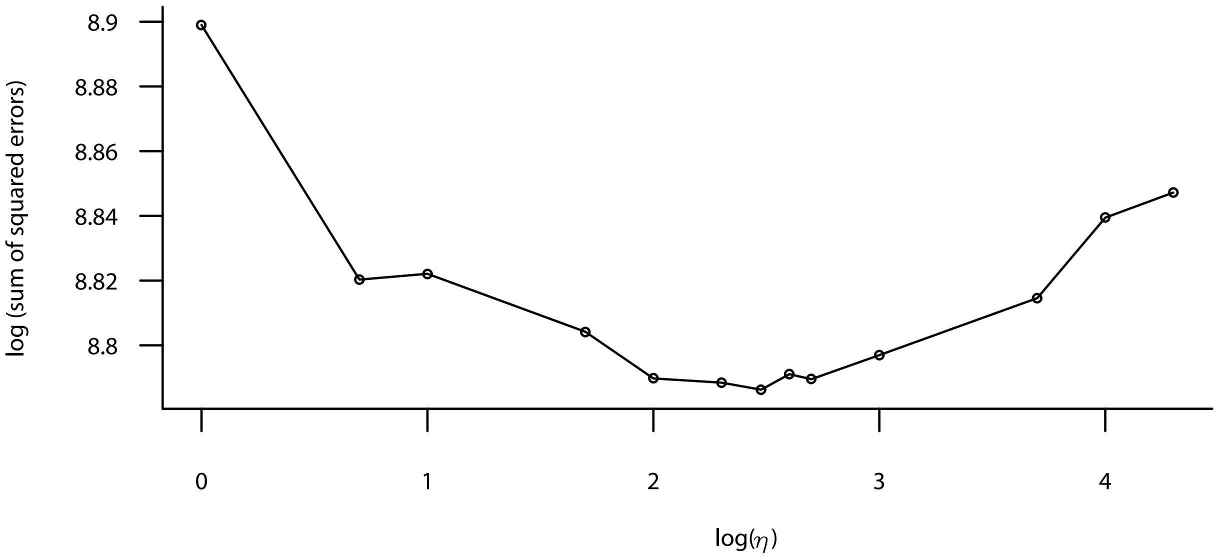

Figure S1.1. Empirical Quadratic Risk on Epidemic Size According to Hyperparameter Value

Higher $\eta-$values yielded much smoother daily effective contact rates, while lower $\eta-$values gave rise to highly variable effective contact rates (see Figure S1.2).

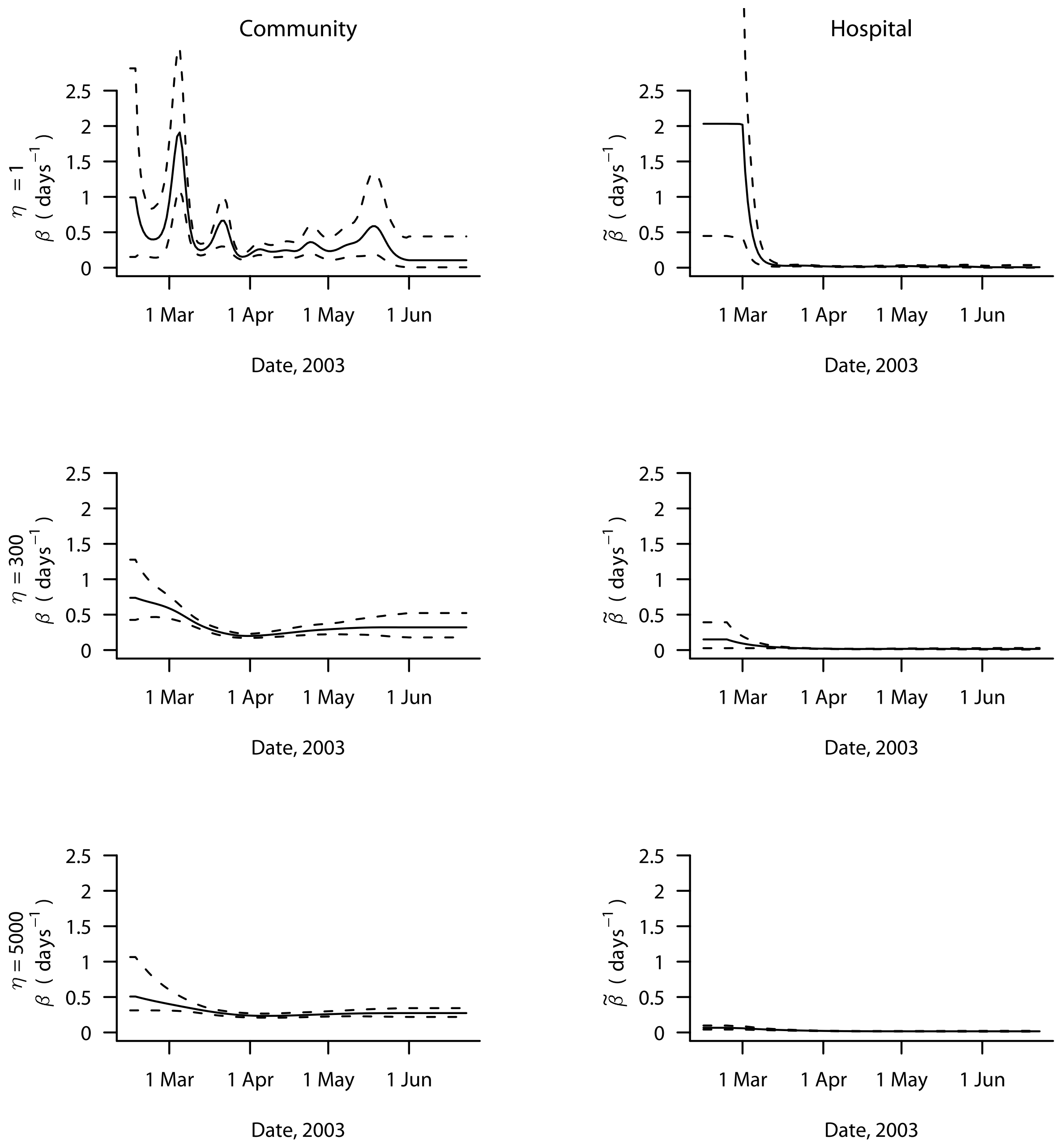

Figure S1.2. Mean Effective Contact Rates (—) and 95% Credible Intervals (- - -) in the Community and Hospitals, as a Function of Time, for Different Values of Hyperparameter η
