# Supplementary material for: Temporal Variability and Social Heterogeneity in Disease Transmission: The Case of SARS in Hong Kong
Source: PLoS Comput Biol. 2009 Aug 21;5(8):e1000471. doi: 10.1371/journal.pcbi.1000471 (PMC2717369; doi:10.1371/journal.pcbi.1000471)
Supplement: Text S2 — Sampler Used for Parameter Estimation (0.07 MB PDF) [file pcbi.1000471.s002.pdf]

Text S2. Sampler Used for Parameter Estimation

At each iteration, the following moves were performed: (a) resampling by blocks of the daily effective contact rates β_t and $\tilde{\beta}_t$ ($t = 1, \ldots, T$, where T is the duration of the epidemic) ; (b) resampling of hyperparameters σ and $\tilde{\sigma}$; (c) for four randomly chosen cases, resampling of the times of onset of infectivity and changing the times for the end of infectivity while keeping the durations of the infectious period constant; (d) resampling of the times for the end of each patient's infectious period ;
(e) resampling of m and q (mean and standard deviation for the duration of the infectious period) ;
(f) resampling of $\left(\tau_1^Y - \tau_0^Y\right) \times \xi^Y$ and $\left(\tau_1^X - \tau_0^X\right) \times \xi^X$ (area under SSE curves); (g) resampling of τ_0^Y and τ_0^X (times SSE started); (h) resampling of $\tau_1^Y - \tau_0^Y$ and $\tau_1^X - \tau_0^X$ (SSE durations).
For move (a), the 'blocking' strategy devised by Knorr-Held was applied [1]. For each block, proposed values were drawn from a multinormal distribution, taking into account temporal correlation. The block size (4 days) was selected to yield approximately 25% acceptance rate. For moves (b) to (h), random-walk Metropolis sampling was performed [2, 3].
Because of the complexity of the sampling, we first checked that, in the absence of data, the posterior joint distribution was not different from the prior distribution. Then 2.1×10^6 iterations of the MCMC were performed with the data, and the first 10^5 were discarded. The output was then sampled every 100 iterations and recorded as an independent sample of size 2^4 from the joint posterior distribution. The computational time was about a week on an IntelXeon X3360 2.8 GHz Linux machine. The convergence of the joint chain was assessed by running chains from various starting points and by visually inspecting the parameter quantiles. There was no indication of failure to converge, although convergence was slower for the first few days of β_t and $\tilde{\beta}_t$ than for other parameters (see Figure S2.1).

References

1. Knorr-Held L (1999) Conditional prior proposals in dynamic models. Scand J Statist 26: 129–144.

2. Gilks W, Richardson S, Spiegelhalter D (1996) Markov chain Monte Carlo in practice. London: Chapman & Hall.

3. Robert C (2001) The Bayesian choice: From decision-theoretic foundations to computational implementation. New York: Springer.

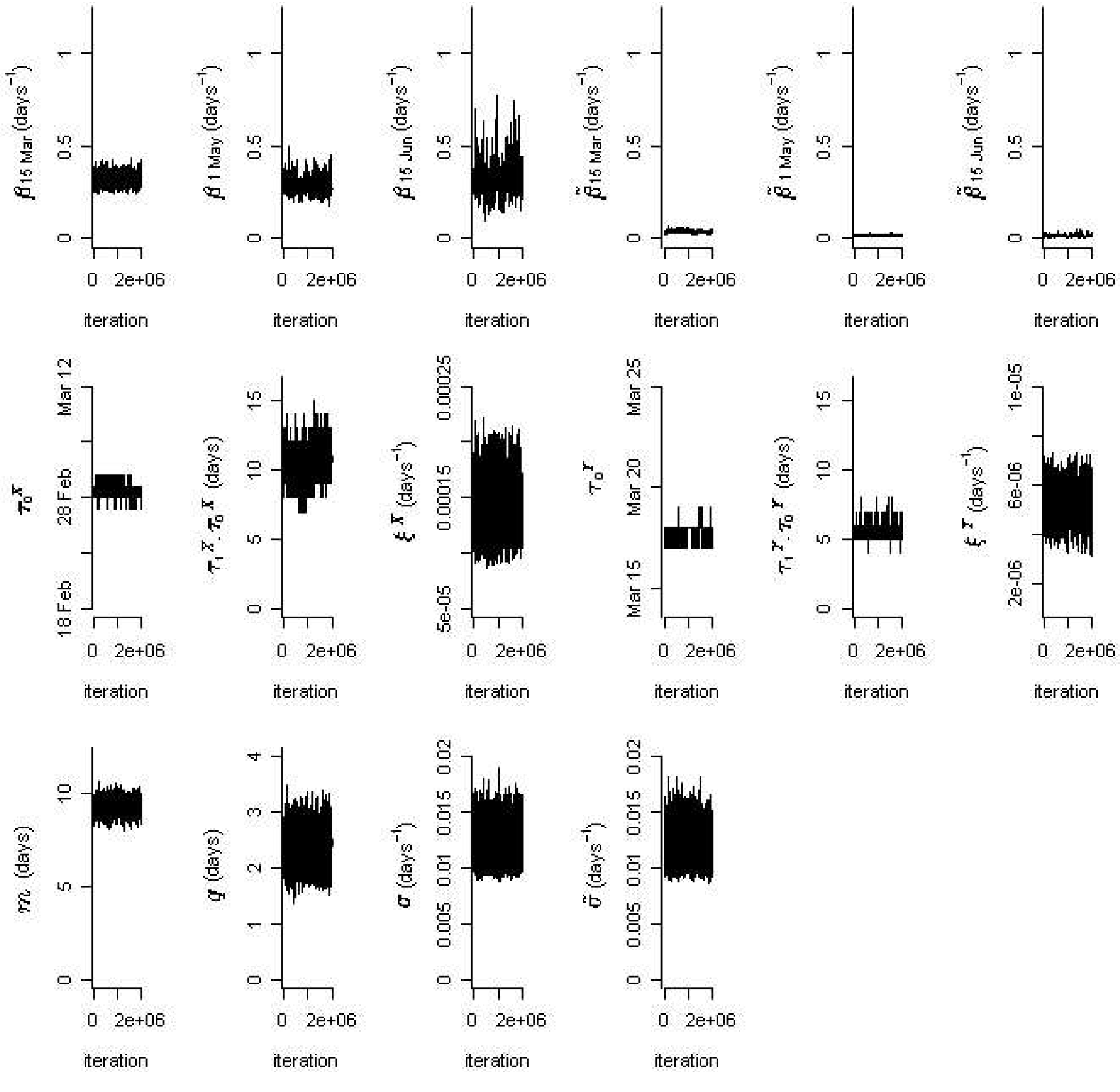

Figure S2.1. Convergence of the Markov Chain
