# Supplementary material for: Temporal Variability and Social Heterogeneity in Disease Transmission: The Case of SARS in Hong Kong
Source: PLoS Comput Biol. 2009 Aug 21;5(8):e1000471. doi: 10.1371/journal.pcbi.1000471 (PMC2717369; doi:10.1371/journal.pcbi.1000471)
Supplement: Text S3 — Estimation of the Case-Reproduction Number RA→Bt (0.39 MB PDF) [file pcbi.1000471.s003.pdf]

Text S3. Estimation of the Case-Reproduction Number $R_t^{A->B}$

In this Appendix, we derive an estimator for the case-reproduction number, $R_t^{A->B}$, the mean number of secondary cases that a category-A infectious individual ($A = gp, hw$, or hp), who was infected on day t, will eventually generate among category-B susceptible individuals ($B = gp, hw$, or hp). In the following, $\mathbf{1}_E(x)$ is the indicator function taking value 1 when $x \in E$ and 0 otherwise.
There are five sources of infection ($g = 1, \ldots, 5$), and three target susceptible compartments ($B = gp, hw, hp$). The matrix $\left(\lambda_t^{g->B}\right)_{g=1,\ldots,5;B=gp,hw,hp}$ of infection rates is defined in Table S3.1.

Table S3.1. Definition of Infection Rates

**Source of infection**	**Target Susceptible Compartments**		
	S^{gp}	S^{hw}	S^{hp}
I^{gp}	$\beta_t \frac{I^{gp}}{N^{gp}+N^{hw}}$	$\beta_t \frac{I^{gp}}{N^{gp}+N^{hw}}$	0
I^{hw}	$\beta_t \frac{I^{hw}}{N^{gp}+N^{hw}}$	$\beta_t \frac{I^{hw}}{N^{gp}+N^{hw}}$	$\tilde{\beta}_t \frac{I^{hw}}{N^{hw}+N^{hp}}$
$H^{gp} \cup H^{hw} \cup H^{hp}$	0	$\tilde{\beta}_t \frac{H^{gp}+H^{hw}+H^{hp}}{N^{hp}}$	$\tilde{\beta}_t \frac{H^{gp}+H^{hw}+H^{hp}}{N^{hw}+N^{hp}}$
Housing Estate Y SSE	$\xi^Y \mathbf{1}_{\left[\tau_0^Y, \tau_1^Y\right]}(t)$	0	0
Hospital X SSE	0	$\xi^X \mathbf{1}_{\left[\tau_0^X, \tau_1^X\right]}(t)$	0

Let m_u^B be the total number of incident cases in compartment S^B on day u, attributed to the five sources of infection. Conditionally on m_u^B, the respective contributions of the five sources of infection have the multinomial distribution with parameters m_u^B, and $(\pi_u^{1->B}, \pi_u^{2->B}, \pi_u^{3->B}, \pi_u^{4->B}, \pi_u^{5->B})$, where $\pi_u^{g->B} = \frac{\lambda_u^{g->B}}{\lambda_u^B}$ for $g = 1, \ldots, 5$. On average, the total number of secondary cases contaminated by patients from the source of infection g on day u in compartment S^B is $m_u^B \pi_u^{g->B}$. Now, as the model is homogeneously mixed, a typical case i from source g on day u is responsible for $\frac{m_u^B \pi_u^{g->B}}{n_u^g}$ secondary cases on average, where n_u^g is the number of cases that are also in source g on day u. Throughout his/her infectious period, given case i thus generates an average of $\sum_{u=\nu_i}^{\psi_i} \frac{m_u^B \pi_u^{g_i(u)->B}}{n_u^{g_i(u)}}$ secondary cases in compartment S^B, where $g_i(u)$ is the source of infection to which i belongs on day u. Averaging over all category-A cases who were infected on day t, our estimator of $R_t^{A->B}$ is $\frac{1}{m_t^A} \sum_{i \in A} \mathbf{1}_{\{\omega_i = t\}} \sum_{u=\nu_i}^{\psi_i} \frac{m_u^B \pi_u^{g_i(u)->B}}{n_u^{g_i(u)}}$.
After the burn-in period, every 100 iterations of the MCMC algorithm, model parameters and augmented data were recorded, and the estimator of $R_t^{A->B}$ was calculated from these values as explained above. This finally gave the posterior distribution of the reproduction number $R_t^{A->B}$, whose mean is represented in Figure S3.1.

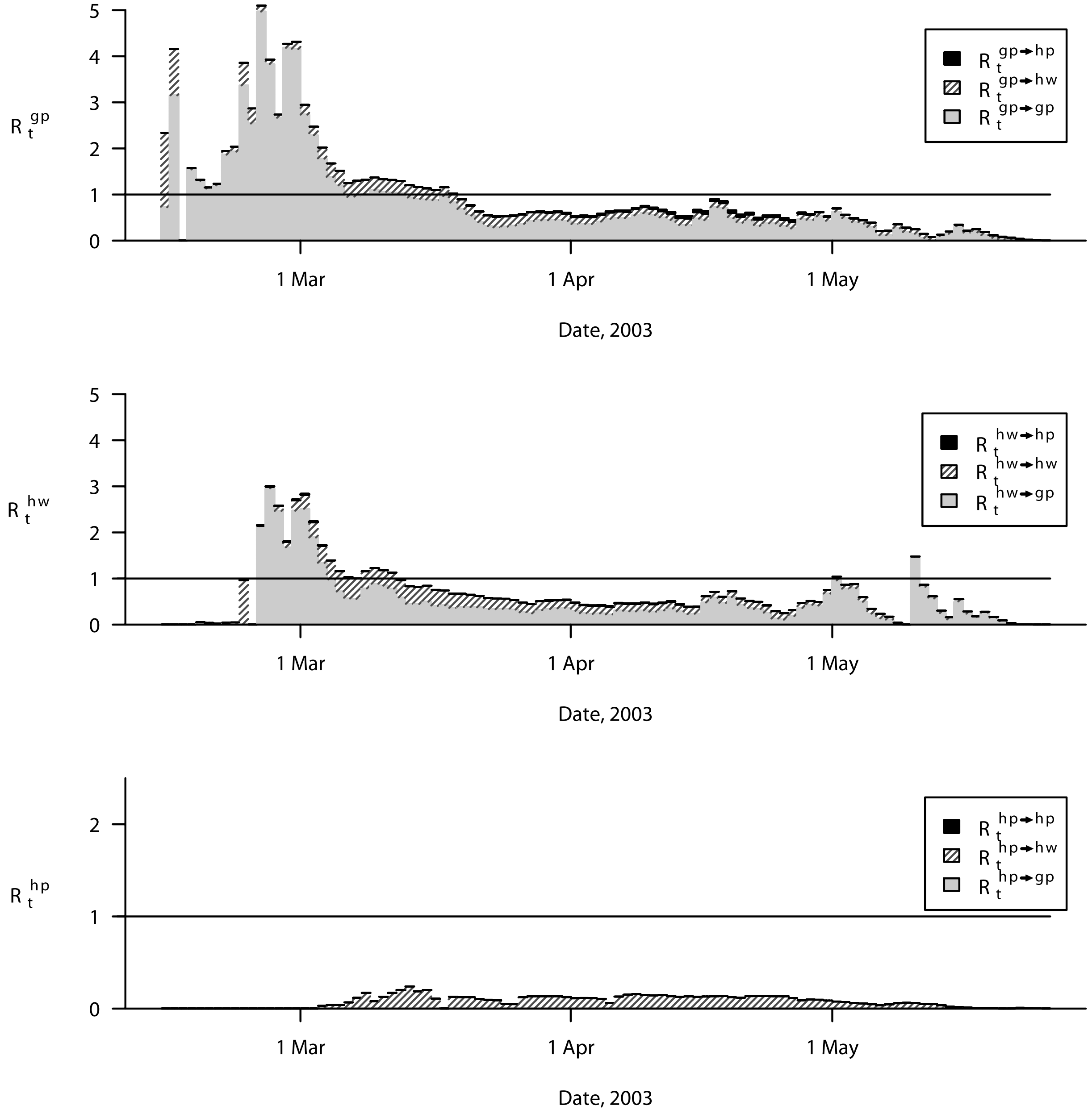

Figure S3.1. Mean Case-Reproduction Numbers in the General Population (R_t^{gp}), among Healthcare Workers (R_t^{hw}) and among Nosocomial Cases (R_t^{hp}) as a Function of Time
$R_t^{A->B}$ is the average number of category-B secondary cases that a category-A patient, infected at time t, will go on to infect, in turn.
