# Supplementary material for: Temporal Variability and Social Heterogeneity in Disease Transmission: The Case of SARS in Hong Kong
Source: PLoS Comput Biol. 2009 Aug 21;5(8):e1000471. doi: 10.1371/journal.pcbi.1000471 (PMC2717369; doi:10.1371/journal.pcbi.1000471)
Supplement: Text S4 — Epidemic Simulation (0.04 MB PDF) [file pcbi.1000471.s004.pdf]

Text S4. Epidemic Simulation

We describe here the procedure used to simulate an epidemic with parameters β_t, $\tilde{\beta}_t$, m, q, ξ^Y, τ_0^Y, τ_1^Y, ξ^X, τ_0^X, τ_1^X in a population of size N including N^{hw} healthcare workers, N^{hp} hospitalized patients and N^{gp} people in the general population, so that $N = N^{gp} + N^{hw} + N^{hp}$. The first infected patient was assumed to belong to the general population. We assumed that his/her symptoms appear on $O_1 =$ February 15, 2003, and he/she is admitted to hospital on $A_1 =$ February 22, 2003. Furthermore, we assumed that he/she dies from SARS ($d_1 = 1$). The time ν_1 when the patient becomes infectious was sampled from a uniform distribution on $\{O_1 - 1, \ldots, O_1 + 4\}$. His/her time of contamination was then chosen as $\omega_1 = \nu_1 - 5$. The duration of infectious period for the first case was drawn from a uniform distribution in $\{0, \ldots, X\}$, where X was sampled from the gamma distribution with mean and standard deviation $(m,\, q)$. The patient's duration of hospital stay was drawn from a gamma distribution with mean and standard deviation as in the dataset.
If k_t new infections have occurred before or on day t, we denote by $\{\omega_1, \ldots, \omega_{k_t}\}$ the times of contaminations $\{\nu_1, \ldots, \nu_{k_t}\}$, the times infectious periods started, $\{O_1, \ldots, O_{k_t}\}$ the times of symptom onset, $\{A_1, \ldots, A_{k_t}\}$ the times of hospital admissions, $\{\psi_1, \ldots, \psi_k\}$ the times infectious periods ended, $\{d_1, \ldots, d_{k_t}\}$ the discharge status ($d_i = 1$ if the patient would die and 0 otherwise), $\{C_1, \ldots, C_{k_t}\}$ each patient's category ($C_i = hw, hp, gp$) and $\{D_1, \ldots, D_{k_t}\}$ the discharge dates.
$S_t^{hw} = N^{hw} - \sum_{i=1}^{k_t} \mathbf{1}_{\{\ C_i\ =\ hw\ \}}$ is the number of susceptible healthcare workers at time t;
$S_t^{hp} = N^{hp} - \sum_{i=1}^{k_t} \mathbf{1}_{\{\ C_i\ =\ hp\ \}}$ is the number of susceptible hospitalized patients at time t; and
$S_t^{gp} = N^{gp} - \sum_{i=1}^{k_t} \mathbf{1}_{\{\ C_i\ =\ gp\ \}}$ the number of susceptible individuals in the general population at time t.
$I_t^C = \sum_{i=1}^{k_t} \mathbf{1}_{\{C_i\ =\ C\}} \mathbf{1}_{\{\nu_i \le t < min(A_i, \psi_i)\}}$ was the number of infectious category-C ($C = gp, hw, hp$) individuals not hospitalized at time t.
$H_t^C = \sum_{i=1}^{k_t} \mathbf{1}_{\{C_i\ =\ C\}} \mathbf{1}_{\{max(\nu_i, A_i) \le t < \psi_i\}}$ was the number of infectious category-C individuals hospitalized at time t.
The number n_{t+1}^C of new category-C individuals infected on day $t+1$ was drawn from a binomial distribution with parameters S_t^C and p_t^C where [1]:

$$\begin{aligned} p_t^{gp} &= 1 - \exp\left(-\lambda_t^{gp}\right) \\ p_t^{hw} &= 1 - \exp\left(-\lambda_t^{hw}\right) \\ p_t^{hp} &= 1 - \exp\left(-\lambda_t^{hp}\right) \end{aligned}$$

where the λ_t^C were defined in the Material and Methods part.
For each case i infected on day $t+1$, $\omega_i = t+1$ and $\nu_i = \omega_i + 5$.
The probability π_{gp} that a case would die was estimated as the observed mortality rate among patients in the general population : $\pi_{gp} = 0.1308$. Similarly, the probability of dying was given by $\pi_{hp} = 0.3977$ for nosocomial SARS cases and by $\pi_{hw} = 0.0228$ for a healthcare worker. For all $i = 1, \ldots, n_t^C$, category C patient i had a probability π_C of dying. If the patient would not die ($d_i = 0$), the duration of the infectious period was drawn from the gamma distribution with mean and standard deviation $(m,\, q)$. If the patient would die ($d_i = 1$), a number X was drawn from the gamma distribution with mean and standard deviation $(m,\, q)$; then, the duration of the infectious period was drawn from a uniform distribution on $\{0, \ldots, X\}$ to take into account the potential reduction of infectious period caused by death. The time of the first symptoms was drawn from a uniform distribution in $\{\nu_i - 4, \ldots, \nu_i + 1\}$. For all non nosocomial cases, the time from symptom onset to hospital admission was drawn from a gamma distribution, with parameters depending on the time of symptom onset, taken from Donnelly et al. [2]. For nosocomial cases, the admission time was arbitrarily

chosen as November 5, 2002. The time of discharge from hospital was Y days after symptoms onset, where Y was sampled from the dataset.

References

1. Lekone PE, Finkenstadt BF (2006) Statistical inference in a stochastic epidemic SEIR model with control intervention: Ebola as a case study. Biometrics 62: 1170–1177.

2. Donnelly CA, Ghani AC, Leung GM, Hedley AJ, Fraser C, et al. (2003) Epidemiological determinants of spread of causal agent of severe acute respiratory syndrome in Hong Kong. Lancet 361: 1761–1766.
